# Bioactive Potentials of Endophytic Fungus *Fusarium concentricum* P2NS8 Derived From *Piper betel* Linn.: Antibacterial, Antioxidant, and Cytotoxic Effects

**DOI:** 10.1155/ijm/7968494

**Published:** 2026-05-30

**Authors:** Ankita Singh, Sita Preedanon, Soottawat Benjakul, Jirayu Buatong

**Affiliations:** ^1^ International Center of Excellence in Seafood Science and Innovation, Faculty of Agro-Industry, Prince of Songkla University, Songkhla, Thailand, psu.ac.th; ^2^ National Biobank of Thailand (NBT), National Science and Technology Development Agency (NSTDA), Pathum Thani, Thailand, nstda.or.th

**Keywords:** antimicrobial activity, endophytic fungi, flavonoid, *Fusarium concentricum*, *P. betel*, piperamide

## Abstract

Natural compounds from microorganisms are gaining importance as food preservation agents to maintain seafood quality and ensure safety. Endophytic fungi from medicinal plants are a crucial microbial resource for producing bioactive compounds. This study is aimed at isolating and identifying the endophytic *Fusarium* spp. from *Piper* spp. plants and determining the antimicrobial activity of their crude extract against spoilage and pathogenic bacteria. The ethyl acetate extract of the endophytic fungus strain P2NS8 (EAE‐P2NS8) exhibited antibacterial activity against *Shewanella* sp. TBRC 5775, *Listeria monocytogenes* ATCC 15313, and *Staphylococcus aureus* ATCC 25923, with MIC and MBC values in the range of 0.128–0.512 mg/mL, 0.256–0.512 mg/mL, and 0.064–0.256 mg/mL, respectively. Scanning electron microscopic (SEM) study revealed significantly damaged cells with wrinkled surfaces and breakage in all three bacterial strains treated with EAE‐P2NS8 (4 × MIC). Additionally, the antioxidant activity of the EAE‐P2NS8 was determined using DPPH‐RSA, ABTS‐RSA, and FRAP assays. The EAE‐P2NS8 at 0.039 mg/mL showed no significant cytotoxicity on Caco‐2 cells. LC‐QTOF‐MS analysis identified kaempferol 3‐(3 ^″^‐acetyl‐6 ^″^‐p‐coumaroylglucoside) and catechin‐4beta‐ol as the most abundant flavonoids in positive and negative modes, respectively. The alkaloid (2E)‐piperamide‐C5:1 was also detected in EAE‐P2NS8 in negative mode. The endophytic fungus strain P2NS8 was identified as *Fusarium concentricum* through morphological and molecular analyses. (2E)‐Piperamide‐C5:1 was first reported to be produced by the endophytic fungus *F. concentricum* isolated from *P. betel*. These findings highlight the potential of secondary metabolites from *F. concentricum* as alternative antimicrobial and antioxidant agents.

## 1. Introduction

Foodborne pathogens are a significant risk to human health and the economy, causing thousands of infections and widespread intestinal disorders. These pathogenic microorganisms can contaminate food products during manufacturing, processing, storage, and shipping [1]. Among these pathogens, bacteria are the most common culprits. To keep food free from harmful microbes, various preservation methods have been developed. Since some foods, especially seafood, are perishable with a short shelf life, proper preservation is required. Spoilage caused by some bacteria brings about the rejection by consumers associated with the economic loss [2]. Apart from microbial spoilage, lipid oxidation could be another critical contributor to the quality loss related with off odor and toxicity of lipid oxidation products [3]. Therefore, effective preservation, particularly through the use of safe additives, is required. However, some synthetic preservatives like sorbates and nitrates can be of health concerns, including allergic reactions [4]. This leads to growing demand for natural compounds, which are proven as safer and even more effective alternatives. Several natural compounds have antimicrobial properties that help ensure food safety.

*Piper betel*, which belongs to the *Piperaceae* family, is one of the extensively growing evergreen tropical plants and a perennial creeper [5, 6]. Due to the presence of strongly fragrant and aromatic compounds, its leaves are commonly used as a mouth freshener in Eastern Asia [7]. Betel, *P. betle* L., commonly known as “phlu” in Thailand, has several applications in traditional medicine [8]. Betel leaves are rich in various antioxidants, including flavonoids, tannins, alkaloids, terpenoids, and saponins. Traditionally, its leaves have been used for their antibacterial and antifungal properties [9]. They are widely used as antibiotics and applied typically to wounds and lesions to promote healing. The antimicrobial properties of betel leaf allow it to inhibit and kill microorganisms, making it an effective preservative [10].

Endophytic fungi are fungi that coexist symbiotically with healthy plant tissues for at least a short period of their life cycles without causing any disease to the plants [11]. Endophytes are essential for helping plants adapt to changing environmental conditions and to overcome stressful conditions. Numerous bioactive substances, including phenol, steroids, saponins, flavonoids, and others, can be produced by endophytic fungi and aid in the host plant′s defense against pathogenic invasion [12]. Some endophytic fungi enhance host plant growth and nutrient uptake while also strengthening the plant′s resistance to pathogens, insects, and herbivores by generating secondary metabolites and stimulating the production of phytoalexins. These fungi have been identified as a promising and novel source of bioactive compounds with numerous applications in the pharmaceutical, food, and agricultural sectors [11]. Endophytic fungi have been shown to produce a variety of biologically active metabolites [13]. Endophytic microorganisms associated with medicinal plants have received augmenting attention recently. These microbes are known to supply numerous unique secondary metabolites, which can be a promising source of medications based on antimicrobial, anticancer, antidiabetic, antioxidant, and immunosuppressive properties [14].

Fungal endophytes, primarily those of the *Fusarium* species, are recognized for their versatile nature. *Fusarium* is a large, widely distributed genus with over 70 species that can produce a diverse range of active metabolites. The remarkable genetic variation within the genus has made *Fusarium* species one of the most significant groups of fungi, influencing not only their biology and interactions with surrounding organisms but also their secondary metabolism [15]. Recently, different species of *Fusarium* have been documented to produce bioactive phytoconstituents exhibiting multifunctional properties, including antiviral, anticancer, antioxidative, antiparasitic, and immunomodulatory effects [14]. There are a few reports on endophytic fungi isolated from *P. betel* [16–18]. However, the antibacterial, antioxidant, and cytotoxic activities of endophytic fungal extracts have not been elucidated. *P. betle* and *P. retrofractum* have a rich history in traditional medicine and are known for their high levels of bioactive compounds. The antibacterial and antifungal properties of the extract from *P. betle* leaves are well documented [7–10]. However, research on the endophytic *Fusarium* associated with these plants is limited, and their potential bioactivities remain largely unexplored. Therefore, this study is aimed at isolating and identifying endophytic fungi from *P. betle* and *P. retrofractum* and evaluating the antibacterial, antioxidant, and cytotoxic activities of their ethyl acetate extracts (EAEs), offering unique and sustainable applications in food safety.

## 2. Materials and Methods

### 2.1. Collection of Plant Materials and Isolation of Endophytic Fungi

The leaves and stems of healthy *P. retrofractum* (P1) and *P. betle* (P2) were collected from Songkhla province, Thailand. A sample of P1 was collected from the Prince of Songkla University, Hat Yai Campus, Songkhla, Thailand, and a sample of P2 was collected from Rattapoom district, Songkhla, Thailand. Fresh plants without any symptoms of plant diseases were used for endophytic fungal isolation.

The leaves were washed with tap water and dried under a laminar flow cabinet. Different parts of the leaves (midrib, lamina, and petiole) and stem were cut into small pieces (1 × 1 cm^2^). For the surface sterilization process, the cut leaf segments were soaked in 95% ethanol for 1 min, followed by immersion of the segments in a chlorine solution (3% v/v) for 5 min. Subsequently, the segments were rinsed with sterile distilled water twice. The sterilized plant segments were dried on the sterilized Petri dish under a laminar flow cabinet. The segments were then placed on Rose Bengal Chloramphenicol Agar plates (RBC) (HiMedia Laboratories Pvt. Ltd, India) and incubated at 25°C–30°C for around 3–7 days. The plates were examined regularly for the growth of fungal mycelium from segments under a stereo zoom microscope (Olympus SZ2‐ST, Japan) every day. The pure culture of endophytic fungi was isolated from plant segments by the hyphal tip isolation technique under a stereo zoom microscope and transferred to grow on potato dextrose agar (PDA) (HiMedia Laboratories Pvt. Ltd, India) [19, 20]. The pure cultures of endophytic fungi were incubated at 25°C–30°C for 3–7 days. The pure cultures were kept in potato dextrose broth (PDB) with glycerol solution (20% v/v) and stored at −80°C for further study.

### 2.2. Characterization and Identification of Endophytic Fungi

#### 2.2.1. Morphological Identification

##### 2.2.1.1. Macroscopic Identification

The endophytic fungi were identified based on their morphology. Macroscopic identification was based on the characteristics of the colony formed on PDA. Characteristics like shape, size, and color were measured following the methods of Barnett and Hunter [21].

##### 2.2.1.2. Microscopic Identification

Microscopic identification included staining the mycelium and spores of the isolate with lactophenol‐cotton blue and observing the spores under the 40× objective. Identification of the isolated fungi was done as described by Barnett and Hunter [21].

### 2.3. Fungal Cultivation and Extraction of Secondary Metabolites From Endophytic Fungi

The endophytic *Fusarium* sp. strains P1LaS7 and P2NS8, representing morphotypes A and B, respectively, were selected for culture and extraction. The endophytic *Fusarium* sp. strains P1LaS7 and P2NS8 were cultured in PDB (300 mL) and incubated at 25^°^C ± 3^°^C under static conditions for 4 weeks. After 4 weeks, the culture broth was separated from the mycelium using a vacuum filter pump with Watman No. 1 filter paper (Cytiva, China). The filtered culture broth was extracted twice with an equal volume of ethyl acetate (Macron, United States). The anhydrous sodium sulfate (Na_2_SO_4_) was added to the extracted ethyl acetate to remove residual water [22]. Then, the ethyl acetate was evaporated using the rotary evaporator (model N‐1000, Tokyo Rikakikai, Co. Ltd, Tokyo, Japan) at 40°C under vacuum. The EAE was flushed with nitrogen gas to remove the solvent completely. Finally, the EAE was stored at 4°C till further experiments were conducted.

### 2.4. Antibacterial Activity Testing of EAE From Endophytic Fungi

#### 2.4.1. Inoculum Preparation

*Staphylococcus aureus* ATCC 25923, *Listeria monocytogenes* ATCC 15313, and *Shewanella* sp. TBRC 5775 were streaked on tryptone soya agar (TSA; OXOID, Basingstoke, United Kingdom) and incubated at 37°C for 18–24 h. Three to five single colonies of bacteria were picked and added into tryptone soya broth (TSB; OXOID, Basingstoke, United Kingdom), incubated at 37°C with continuous shaking at 180 rpm/min for 3–5 h. After incubation, sterile 0.85% normal saline solution (NSS) was used to adjust bacterial turbidity to 0.5 McFarland standard (MF), and the turbidity was measured using Grant bio DEN‐1 (Grant instruments Ltd, Cambs, England).

#### 2.4.2. Determination of Minimal Inhibitory Concentration (MIC) and Minimal Bactericidal Concentration (MBC) of EAE

The MICs of EAE were determined using the colorimetric broth microdilution method according to a modification of CLSI MA7‐A4 (CLSI, 2002a). The EAE was dissolved in dimethyl sulfoxide (DMSO; Sigma‐Aldrich, Co., United States) to prepare stock solutions of 100 mg/mL and stored at 4°C until use. The EAE stock was diluted in Muller‐Hinton broth (MHB; OXOID, Basingstoke, United Kingdom) in a 96‐well polystyrene microplate (SPL Life Sciences, Pocheon‐si, Korea) using a two‐fold serial dilution method in a volume of 50 *μ*L. The bacterial inoculum (0.5 MF) was diluted with MHB at a ratio of 1:200 to obtain ~7.5 × 10^5^ CFU/mL of inoculum, and then the inoculum (50 *μ*L) was added to each well. The final concentration of EAE ranged from 0.004 to 4.096 *μ*g/mL. Plates were incubated at 37°C for 15 h, and then 20 *μ*L of resazurin indicator (0.018% w/v) was added to each well and examined after incubation for 2–3 h at 37°C [23]. The MHBN supplemented with 2% (v/v) DMSO was employed as a negative control [24]. Potassium sorbate at a final concentration ranging from 0.25 to 128 mg/mL was used as a positive control for *Shewanella* sp. TBRC 5775 and nisin for *L. monocytogenes* ATCC 15313 and *S. aureus* ATCC 25923. The lowest concentration of EAE, which inhibited bacterial growth (blue or purple color), was recorded as the MIC value. The MBC value was determined by the drop plate method. The concentrations of EAE higher than or equal to the MIC value were dropped onto the Muller‐Hinton agar (MHA; OXOID, Basingstoke, United Kingdom). The MHA plates were incubated at 37°C for 24 h. The lowest concentration of extract that killed organisms (no growth) was recorded as the MBC [23].

### 2.5. Scanning Electron Microscopic (SEM) Observation

The inoculum of *S. aureus* ATCC 25923, *L. monocytogenes* ATCC 15313, and *Shewanella* sp. TBRC 5775 was prepared with NSS to obtain 1.5 × 10^8^ CFU/mL and diluted with TSB medium to obtain ~5 × 10^5^ CFU/mL. Five hundred microliters of each inoculum was treated with 4 × MIC of EAE of strain P2NS8 (EAE‐P2NS8) and incubated at 37°C for 24 h. The inoculum cultured in TSB medium containing 2% (v/v) DMSO was used as a negative control. After incubation, the cell pellet was harvested by centrifugation at 3770 g for 10 min. The supernatant was discarded, and the cell pellet was resuspended in sterile phosphate buffer solution (PBS, 0.1 M, pH 7.2). The cell suspension (10 *μ*L) was applied to a glass slide coated with poly‐L‐lysine (Electron Microscopy Sciences, Hatfield, PA, United States) and dried under laminar flow for 30 min. The adherent cells on the cover glass were fixed with 2.5% glutaraldehyde in a 24‐well plate for 2 h at room temperature. The 2% glutaraldehyde was then discarded and gently washed with 0.1 M PBS (pH 7.2), followed by sterile distilled water three times. The fixed cells were then dehydrated using a series of ethanol (50%–100%) and then critical‐point dried. The samples were then sputter‐coated with gold for 1 min and visualized using an FEI Quanta 400 Scanning Electron Microscope (FEI Czech Republic, Brno, Czech Republic). The morphological changes in bacterial cells (holes or distortion on the cell surface) were determined [25].

### 2.6. Determination of Total Phenolic Content (TPC) of EAE From Endophytic Fungus P2NS8

The TPC of EAE‐P2NS8 was examined using Folin–Ciocalteu′s reagent (FCR). One hundred microliters of EAE‐P2NS8 solution (10 mg/mL) was mixed with FCR (750 *μ*L), followed by 6% sodium carbonate (750 *μ*L) in a 96‐well polystyrene microplate in triplicate. Absorbance was read at 760 nm. Gallic acid was used as a standard for phenolic compounds in this study. The TPC was calculated and reported as mg gallic acid equivalent (GAE)/g of EAE [26].

### 2.7. Determination of Total Flavonoid Content (TFC) of EAE From Endophytic Fungus P2NS8

The TFC of EAE‐P2NS8 was determined using the colorimetric assay as described by Tagrida and Benjakul [8]. The EAE‐P2NS8 solution (200 *μ*L, 10 mg/mL) was reacted with 5% (w/v) sodium nitrite (60 *μ*L) and 10% (w/v) aluminum chloride (60 *μ*L), and 1 M NaOH (400 *μ*L) in a 96‐well polystyrene microplate in triplicate. Absorbance was measured at 510 nm. The TFC was reported as mg quercetin equivalent (QE)/g of EAE.

### 2.8. Determination of Antioxidant Activities of EAE From Endophytic Fungus P2NS8

#### 2.8.1. 2,2‐Diphenyl‐1‐Picrylhydrazyl (DPPH) Radical Scavenging Activity

DPPH‐RSA was determined following the method of Tagrida and Benjakul [8]. Briefly, EAE‐P2NS8 solution (300 *μ*L, 10 mg/mL) was mixed with DPPH solution in 2700 *μ*L of methanol (0.15 mM) in a 96‐well polystyrene microplate in triplicate. The microplate was then incubated in the dark at 25°C for 60 min. Absorbance at 517 nm was observed. The results were expressed as *μ*mol Trolox equivalents (TE)/g of EAE.

#### 2.8.2. Azino‐bis (3‐Ethylbenzothiazoline‐6‐Sulphonic Acid) (ABTS) Radical Scavenging Activity

ABTS‐RSA (150 *μ*L, 10 mg/mL) was mixed with ABTS solution (2850 *μ*L) in a 96‐well polystyrene microplate in triplicate, and the mixture was incubated for 1 h in the dark at room temperature. Absorbance was measured at 734 nm. Distilled water was used as a blank for the samples. The Trolox standard curve (0–600 *μ*M) was used for the calculation of activities. The results were expressed as *μ*mol TE/g of EAE [26].

#### 2.8.3. Ferric Reducing Antioxidant Power (FRAP)

FRAP was determined following the method of Benjakul [26]. Briefly, EAE‐P2NS8 solution (150 *μ*L, 10 mg/mL) was mixed with 2.85 mL of working FRAP reagent in a 96‐well polystyrene microplate in triplicate. The reaction mixture was incubated for 30 min in the dark conditions at room temperature. Absorbance was measured at 593 nm, and the results were expressed as *μ*mol TE/g of EAE.

### 2.9. Cytotoxicity Assay of EAE From Endophytic Fungus P2NS8

The cytotoxicity of EAE‐P2NS8 was evaluated using 3‐(4, 5‐dimethylthiazolyl‐2‐yl)‐diphenyl‐tetrazolium bromide (MTT) assay [27]. The human colon carcinoma cell line (Caco‐2, ATCC: HTB‐37, United States) was cultured in 96‐well culture plates containing Eagle′s Minimum Essential Medium (EMEM, Gibco, United States) containing 10% fetal bovine serum (FBS, Gibco, United States) and antibiotics (100 U penicillin and 100 U/mL streptomycin, Gibco, United States) and incubated at 37°C in 5% CO_2_ incubator. Trypsinization was performed using 0.25% trypsin‐EDTA (Gibco, United States), followed by the addition of fresh culture medium to create a new single cell suspension for further incubation. The Caco‐2 cell line (1 × 10^5^ cells/mL) was added to a 96‐well plate containing culture medium. After 24‐h incubation, the medium was discarded and replaced with fresh medium containing EAE‐P2NS8 at various concentrations, and phosphate‐buffer saline was used as a negative control. The 96‐well plate was incubated at 37°C in 5% CO_2_ for 24 h. The medium was discarded, and the 20 *μ*L of MTT solution (5 mg/mL) was added to the wells along with the fresh medium (80 *μ*L), followed by incubating at 37°C under 5% CO_2_ for 4 h. Thereafter, the media were removed, and DMSO (100 *μ*L) was added. The absorbance was measured with a microplate reader (Biohit 830, Biohit, Helsinki, Finland) at 570 nm. The cell viability of Caco‐2 cells was calculated based on the absorbance, following this formula:

Cell viability%=At/Ac×100

where *A*_*t*_ and *A*_*c*_ are the absorbances for treated and control cells, respectively. The cytotoxic effect was evaluated by measuring their cell viability.

### 2.10. Molecular Identification of Endophytic Fungus P2NS8

Endophytic fungus strain P2NS8 was cultured for 7 days in PDB at 25°C. The mycelium was harvested and stored in −80°C until further use. DNA was extracted from the mycelia using the CTAB method [28]. Five loci, namely partial sequences of the translation elongation factor 1‐alpha (*tef1-α*), RNA polymerase largest subunit (*rpb1*), beta‐tubulin (*tub2*), internal transcribed spacer (ITS) rDNA, and RNA polymerase second largest subunit (*rpb2*), were amplified using the T100TM Thermal Cycler (BIO‐RAD Laboratories, Inc., Hercules, CA, United States). Primer pair and PCR amplification procedures were described [29–31]. The sample was amplified in a total volume of 50 *μ*L. PCR reaction mixture contained Taq DNA polymerase enzyme (Thermo Fisher Scientific Inc., Waltham, MA, United States) (0.2 *μ*L), each primer (10 *μ*M/*μ*L; 1 *μ*L), genomic DNA (100 ng/*μ*L; 1 *μ*L), dNTPs (1 *μ*L), PCR buffer with (NH_4_)_2_SO_4_ (5 *μ*L), 25 mM MgCl_2_ (5 *μ*L), and milliQ water (35.8 *μ*L). The quality and integrity of the resulting PCR products were checked on a 1% agarose gel using RedSafeTM Nucleic Acid Staining Solution (20,000×) and visualized under an ultraviolet transilluminator. The PCR products were purified and sequenced by Macrogen Inc. (Seoul, South Korea). Multiple sequence alignments were analyzed according to Yilmaz [31] and Preedanon [32]. The assembly sequences were performed through BioEdit 7.2.5 [33] and aligned with Muscle 3.8.31 [34]. The phylogenetic tree and bootstrap analyses were constructed based on combined sequences from five gene regions using the maximum parsimony (MP) method with the aid of the PAUP∗ program (Version 4.0a) and the maximum likelihood (ML) method using the CIPRES web portal [35] through RAxML 8.2.4 [36]. The BFGS method was adopted to optimize GTR rate parameters. The DNA sequences were directly submitted to the NCBI GenBank database for accession numbers and identification.

### 2.11. Identification of Bioactive Compounds in EAE Using Liquid Chromatography‐Quadrupole Time‐of‐Flight Mass Spectrometry (LC‐QTOF‐MS) analysis

The EAE‐P2NS8 was analyzed using LC‐QTOF‐MS, Agilent 1290 Infinity II LC‐6545 Quadrupole‐TOF (Agilent Technologies, Waldbronn, Germany). The EAE‐P2NS8 was dissolved in deionized water (10 mg/mL), centrifuged at 10,000 × g for 5 min, and then filtered through a 0.2‐*μ*m nylon membrane. The identification of bioactive compounds was analyzed following the method by [37]. The possible compounds were identified qualitatively based on their molecular ion peaks, mass‐to‐charge ratios (m/z), and fragment ions. The compounds were identified using the Mass Hunter METLIN database PCD (Personal Compound Database) and PCDL (Personal Compound Database and Library) Version 8 from Agilent Technologies.

## 3. Results and Discussion

### 3.1. Isolation, Characterization, and Identification of Potential Endophytic Fungi

A total of 124 isolates of endophytic fungi were isolated from 32 segments of the midrib, lamina, petiole, and stem of *P. retrofractum* (P1) and *P. betel* (P2) (Table 1). Out of 124 isolates, seven isolates were identified as *Fusarium* spp. based on morphological characteristics. The seven *Fusarium* isolates were classified into two distinct morphotypes (morphotypes A and B) (Table S1).

**Table 1:** Endophytic fungi isolated from different parts of *P. retrofractum* and *P. betle* leaves.

Plant	Number of endophytic fungi (isolates)				Total
	Midrib	Lamina	Petiole	Stem	
*P. retrofractum*	13	10	0	0	23
*P. betle*	16	26	18	41	101
Total	29	36	18	41	124

**Figure 1:**
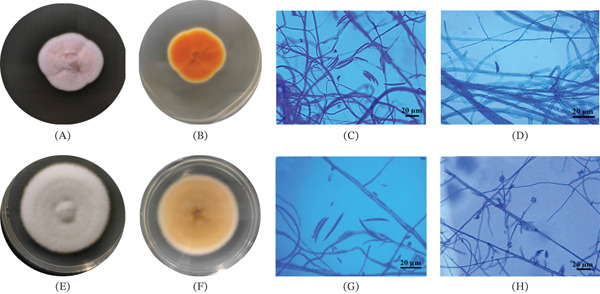
Morphological characteristics of the endophytic fungus strains (A–D) P1LaS7 and (E–H) P2NS8 after 7 days on PDA: (A, E) front colony, (B, F) back colony, (C, D, G) macroconidia, and (H) microconidia. Scale bar, 20 *μ*m.

The strain P1LaS7, representing morphotype A, exhibited a pale orange to white color on the upper surface and a dark orange on the lower surface of the colony. A cottony mycelium in texture with abundant aerial mycelium was observed, and lobulated margins on the PDA plate occurred after 7 days of incubation (Figure 1A,B). The macroconidia were hyaline with two to three septa, sickle‐shaped, 24.8–63.6 *μ*m in length, and 2.4–4.7 *μ*m in diameter (Figure 1C,D).

After a 7‐day incubation on a PDA plate, the strain P2NS8, representing morphotype B, displayed a colony surface that was white to pale purple, flat or slightly elevated at the center, and exhibited irregular, filiform, and alternating pale purple rings on the reverse side (Figure 1E,F). The macroconidia were hyaline with three to four septa, sickle‐shaped, 20.3–34 *μ*m in length, and 1.9–3.2 *μ*m in diameter (Figure 1G). The microconidia in the aerial mycelium were oval, nonseptate, and ranged in diameter from 2.1 to 2.9 *μ*m and in length from 5.1 to 9.6 *μ*m (Figure 1H). Endophytic *Fusarium* spp. isolated from the medicinal plants are promising for producing a variety of bioactive compounds with antimicrobial activity [38]. Endophytic *Fusarium* spp. isolated from *Piper nigrum* has been reported as a dominant species [39]. The endophytic fungus *Fusarium* sp. strains P1LaS7 and P2NS8 were selected for culturing in PDB medium. Thereafter, secondary metabolites were extracted from the filtered culture broth, where EAEs were obtained.

### 3.2. Antibacterial Activity of EAE Against Different Spoilage and Pathogenic Bacteria

The MIC and MBC values of EAEs from selected four endophytic *Fusarium* species against pathogenic and spoilage bacteria have been provided in Table S2. The EAE of *Fusarium* sp. strain P1LaS7 showed antibacterial activity against *L. monocytogenes* ATCC 15313 and *S. aureus* ATCC 25923 at MIC values of 1.024 and 0.512 mg/mL, respectively (Table 2). The EAE of *Fusarium* sp. strain P2NS8 extract showed higher antibacterial activity against all three tested bacteria, including *Shewanella* sp. TBRC 5775, *L. monocytogenes* ATCC 15313, and *S. aureus* ATCC 25923, with MIC values of 0.128, 0.256, and 0.064 mg/mL, respectively (Table 2). A wide range of antimicrobial activity has been shown by crude extracts of numerous *Fusarium* sp., including *Fusarium solani* from *Taxus baccata*, *Fusarium equiseti* from *Garcinia parvifolia*, *Fusarium oxysporum* from *Chromolaena odorata*, and *Fusarium lateritium* from *Rhizophora mucroata* [15]. Low MIC values for the EAE‐P2NS8 indicated broad‐spectrum antibacterial activity against Gram‐positive and Gram‐negative bacteria. Earlier studies reported that the endophytic fungus *Fusarium* sp. showed antimicrobial activity against *S. aureus*, *Klebsiella pneumoniae*, and *Escherichia coli* [38]. However, the extract from the endophytic fungus *Fusarium* sp. rarely exhibited antibacterial activity against *Shewanella* sp. and *L. monocytogenes*. The endophytic fungus *Fusarium* sp. strain P2NS8 was specifically chosen for further investigation because it exhibited significantly higher and broader spectrum antibacterial activity than the other isolate. Additionally, EAE‐P2NS8 exhibited lower MICs against all three tested bacteria. Therefore, it is the most promising candidate for exploring antimicrobial mechanisms.

**Table 2:** Minimum inhibitory concentration and minimum bactericidal concentration of EAE from endophytic fungi *Fusarium* species against pathogenic bacteria and spoilage bacteria.

Bacterial strains	MIC/MBC (mg/mL)			
	P1LaS7	P2NS8	Potassium sorbate	Nisin
SP	> 4.096/> 4.096	0.128/0.512	8/16	—
LM	1.024/2.048	0.256/0.512	—	0.5/1
SA	0.512/1.024	0.064/0.256	—	0.25/0.5

Abbreviations: LM: *L. monocytogenes* ATCC 15313; MBC: minimum bactericidal concentration; MIC: minimum inhibitory concentration; SA: *S. aureus* ATCC 25923; SP: *Shewanella* sp. TBRC 5775.

### 3.3. Effect of EAE‐P2NS8 on the Morphology Change of Bacterial Cells

**Figure 2:**
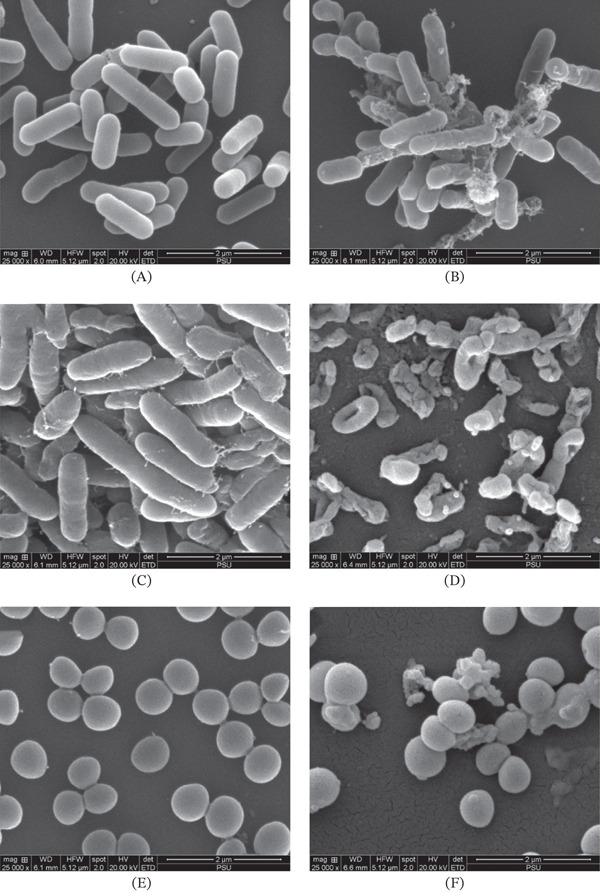
Scanning electron micrograph of untreated cells of (A) *L. monocytogenes* ATCC 15313, (C) *Shewanella* sp. TBRC 5775, and (E) *S. aureus* ATCC 25923, and the treated cells of (B) *L. monocytogenes* ATCC 15313, (D) *Shewanella* sp. TBRC 5775, and (F) *S. aureus* ATCC 25923 with EAE from endophytic fungus strain P2NS8 at the concentration of 4 × MIC.

EAE‐P2NS8, with the highest antimicrobial activity, was chosen for the investigation of its mode of action against bacterial cells. The effect of EAE‐P2NS8 (4 × MIC) on the cell morphology was examined using SEM. Based on SEM images, untreated cells of *L. monocytogenes* ATCC 15313, *Shewanella* sp. TBRC 5775, and *S. aureus* ATCC 25923 exhibited smooth cell surfaces, as illustrated in Figure 2A,C,E, respectively. On the other hand, the cells of *L. monocytogenes* ATCC 15313, *Shewanella* sp. TBRC 5775, and *S. aureus* ATCC 25923 treated with the EAE‐P2NS8 at the concentration of 4 × MIC for 24 h had completely deformed, crumbled, and structurally broken features (Figure 2B,D,F). The smooth rod‐shaped bacteria of *L. monocytogenes* ATCC 15313 were observed (Figure 2A), whereas the treated cells of *L. monocytogenes* ATCC 15313 are completely deformed, crumbled, and structurally broken (Figure 2B). The untreated rod‐shaped cell was found in *Shewanella* sp. TBRC 5775 (Figure 2C), whereas Figure 2D shows the treated cells of *Shewanella* sp. TBRC 5775, which had some pores in the structure and injured and abnormal cells. The untreated spherical‐shaped *S. aureus* ATCC 25923 was noticeable (Figure 2E), whereas the treated cells of *S. aureus* ATCC 25923 showed destruction of some cells with a change in morphology (Figure 2F). The cells in all treated samples generally exhibited morphological changes, such as shrinkage and wrinkling. Cells not only underwent shrinkage but also showed the formation of cavities in the cell outer layer, which might cause cytoplasmic leakage. Furthermore, the cells were completely ruptured and deformed, leading to the loss of cellular material and to their complete lysis. The EAE‐P2NS8 exhibited bactericidal effect against various bacterial strains across different concentrations. This might be due to the presence of kaempferol 3‐(2 ^″^,3 ^″^‐diacetyl‐4 ^″^‐p‐coumaroylrhamnoside), which was abundant in EAE‐P2NS8. Kaempferol 3‐(2 ^″^,3 ^″^‐diacetyl‐4 ^″^‐p‐coumaroylrhamnoside) is a flavonoid glycoside containing three hydroxyl groups (–OH) on the flavonol backbone linked to acylated rhamnoside residues (2 ^″^,3 ^″^‐diacetyl‐4 ^″^‐p‐coumaroylrhamnopyranoside) [40]. Hydroxyl groups (–OH) on the flavonol backbone can form hydrogen bonds with the polar head groups of phospholipids on bacterial cell membranes, leading to cell membrane disruption and the leakage of cytoplasmic contents [41]. The antibacterial activity of kaempferol 3‐(2 ^″^,3 ^″^‐diacetyl‐4 ^″^‐p‐coumaroylrhamnoside) has never been reported. However, kaempferol glycoside derived from *Herissantia tiubae* has been shown to exhibit antibacterial activity against *S. aureus*, with a minimum inhibitory concentration (MIC) of 256 *μ*g/mL. Additionally, this compound has been reported to inhibit a putative efflux pump in bacterial cells, leading to accumulation of effective compounds within the cells [42]. This mechanism can enhance the activity of other active compounds in combination. Furthermore, diosmetin was identified in EAE‐P2NS8 and may contribute to the antibacterial activity. Diosmetin has been reported to inhibit pyruvate kinase enzyme in methicillin‐resistant *Staphylococcus aureus* (MRSA) [43]. Pyruvate kinase is a catalytic enzyme in the glycolysis pathway of bacteria used for the conversion of adenosine diphosphate (ADP) and phosphoenolpyruvate to adenosine triphosphate (ATP) and pyruvate, respectively [44]. These compounds were identified in EAE‐P2NS8 and may have acted synergistically to enhance antibacterial activity by targeting distinct bacterial mechanisms.

### 3.4. Total Phenolic and TFCs of EAE‐P2NS8

Flavonoids and phenolics significantly reduce oxidative stress because they are excellent scavengers of oxidizing chemicals and free radicals, which are linked to several conditions [45]. In addition to their many health advantages, phenolic chemicals are well‐known for their antibacterial properties [46]. EAE‐P2NS8 showed notable differences in TPC and TFC, as tabulated in Table 3. EAE‐P2NS8 had TPC of 23.87 ± 1.85 mg GAE/g of EAE. TPC, which is derived from the sum of flavonoids, phenolic acids, and other polyphenols, was related with antioxidant ability [47]. EAE‐P2NS8 showed very high TFC 1298.89 ± 1.97 mg quercetin/g EAE. TFC reflected antioxidant capacity based solely on flavonoid (a subgroup of phenolic compounds) composition [47]. TFC of EAE‐P2NS8 accounted for more than 80%–90% of TPC. This suggested that flavonoids were the major compounds among all the compounds.

**Table 3:** Total phenolic content, total flavonoid content, and antioxidant activity of EAE from endophytic fungus *F. concentricum* strain P2NS8.

Parameter	Content
TPC (mg GAE/g EAE)	23.87 ± 1.85
TFC (mg QE/g EAE)	1298.89 ± 1.97
DPPH‐RSA (*μ*mol TE/g EAE)	21.78 ± 1.97
ABTS‐RSA (*μ*mol TE/g EAE)	299.52 ± 1.57
FRAP (*μ*mol TE/g EAE)	35.75 ± 3.09

*Note:* Data are represented as mean ± SD (*n* = 3).

Abbreviations: ABTS, 2,2‐azinobis‐(3‐ethylbenzothiazoline‐6‐sulfonic acid) diammonium salt‐radical scavenging activity; DPPH, 2,2diphenyl 1‐picrylhydrazyl‐radical scavenging activity; FRAP, ferric reducing antioxidant power; TFC, total flavonoid content; TPC, total phenolic content.

### 3.5. Antioxidant Activities of EAE‐P2NS8

Endophytic fungi are renowned sources of naturally occurring chemical compounds with antioxidant properties [48]. Antioxidant activities of EAE‐P2NS8 are presented in Table 3. The samples′ antioxidant activity, measured as *μ*mol TE/g of dry solid, varied by assay and sample. EAE‐P2NS8 had the ABTS‐RSA value of 299.52 ± 1.57 * μ*mol Trolox/g EAE. ABTS radical scavenging activity of EAE‐P2NS8 indicated that it can scavenge free radicals and donate protons in an aqueous solution. Because ABTS is soluble in both organic and aqueous media, it can be used to assess the radical‐scavenging capabilities of both lipophilic and hydrophilic molecules [49]. The DPPH‐RSA of EAE‐P2NS8 was found to be 21.78 ± 1.97 * μ*mol Trolox/g EAE. In contrast, DPPH is often analyzed in an organic solvent such as methanol or ethanol. Because DPPH can only dissolve in organic media, especially alcoholic solutions, and not in aqueous solutions, DPPH′s capacity to evaluate hydrophilic antioxidants is limited [50]. FRAP values for EAE‐P2NS8 were 35.75 ± 3.09  *μ*mol Trolox/g EAE. FRAP measures its ability to transfer electrons to ferric ions, thereby forming ferrous counterparts [26].

### 3.6. Cytotoxicity Test of EAE‐P2NS8

**Figure 3:**
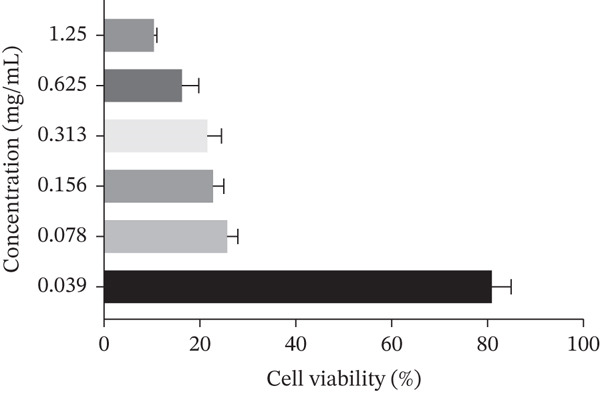
Cytotoxicity of EFE from endophytic fungus strain P2NS8. The Caco‐2 cells were treated with EFE at different concentrations. Error bars represent standard deviation (*N* = 3).

The cytotoxicity of EAE‐P2NS8 on Caco‐2 cells, assessed by the MTT assay, is shown in Figure 3. The result showed that EAE‐P2NS8 at a concentration of 0.039 mg/mL yielded cell viability exceeding 80% (Figure 3). Moreover, EAE‐P2NS8 concentrations above 0.039 mg/mL induced significant cytotoxicity, with cell viability below 70%. As the concentration of EAE‐P2NS8 increased, a decrease in cell viability was observed. These results revealed a pronounced dose‐dependent relationship, with cell viability declining as the EAE‐P2NS8 concentration increased. Specifically, at higher concentrations, particularly those exceeding 0.078 mg/mL, cell viability was lowered to around 30%, indicating a toxic effect. In a previous study, the cytotoxic effects of mycotoxins produced by *Fusarium* species were assessed [51]. investigated the cytotoxicity of beauvericin (BEA) and fusaproliferin (FUS) on human Caco‐2 cells. The results demonstrated that BEA and FUS exhibited dose‐dependent cytotoxicity in Caco‐2 cells, as measured by the MTT assay. This decrease in viability suggested that EFE at elevated levels could be detrimental to health and complicate its application. Further investigations into the mechanism of toxicity and the specific components of the EAE‐P2NS8 responsible for this effect are needed to better understand how to utilize this extract safely and effectively.

### 3.7. Identification of Compounds in EAE‐P2NS8

**Figure 4:**
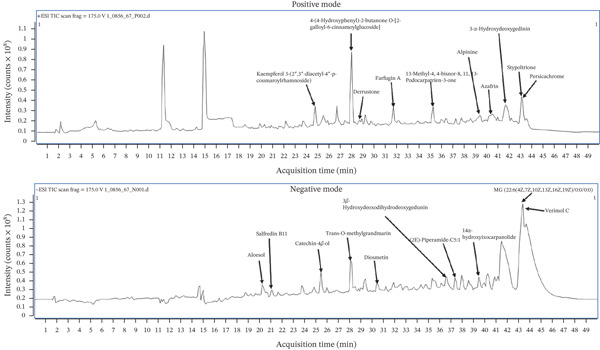
Chromatogram with major peaks of EAE from endophytic fungus *F. concentricum* P2NS8 in positive and negative modes using LC‐QTOF‐MS analysis.

LC‐QTOF‐MS analysis of EAE‐P2NS8 was performed using the positive and negative ionization modes of MS. The 10 compounds with the highest peaks in both positive and negative modes and a matching score greater than 90% were selected (Table 4). The chromatograms for the peaks in both negative and positive modes are illustrated in Figure 4. In positive mode, the highest peak was observed for 4‐(4‐hydroxyphenyl)‐2‐butanone O‐[2‐galloyl‐6‐cinnamoylglucoside], which was dominant with the height of 9.19 × 10^6^, followed by stypoltrione, azafrin, and farfugin A, respectively. However, kaempferol (3,4 ^′^,5,7‐tetrahydroxyflavone, the major compound in the positive mode of EAE‐P2NS8) is a natural flavanol related to the flavonoid group and has been reported as an effective bioactive compound with antimicrobial, anticancer, antioxidant, and anti‐inflammatory effects [52].

**Table 4:** Identification of major components in EAE from endophytic fungus *F. concentricum* strain P2NS8 using LC‐QTOF‐MS in positive and negative modes.

Identified compounds	Class of compounds	Formula	*m/z*	Mass	*R*_*t*_(min)	Height (×10^6^)
Positive mode						
4‐(4‐Hydroxyphenyl)‐2‐butanone O‐[2‐galloyl‐6‐cinnamoylglucoside]	Glycoside	C_32_ H_32_ O_12_	631.1805	608.1913	28.032	9.19
Stypoltrione	Diterpenoid	C_27_ H_36_ O_4_	425.2681	424.2607	43.085	3.44
Azafrin	Sesquiterpenoid	C_27_ H_38_ O_4_	427.286	426.2787	41.767	3.24
Farfugin A	Sesquiterpenoid	C_15_ H_18_ O	237.125	214.1358	31.723	2.76
13‐Methyl‐4,4‐bisnor‐8,11,13‐podocarpatrien‐3‐one	Diterpenoid	C_16_ H_20_ O	251.1407	228.1514	35.239	2.73
Kaempferol 3‐(2 ^″^,3 ^″^‐diacetyl‐4 ^″^‐p‐coumaroylrhamnoside)	Flavonoid	C_34_ H_30_ O_14_	663.1707	662.1633	24.756	2.67
Persicachrome	Diterpenoid	C_25_ H_36_ O_3_	385.2754	384.2681	43.085	2.36
Derrustone	Isoflavonoid	C_18_ H_14_ O_6_	327.0856	326.0783	28.057	1.93
Alpinine	Alkaloid	C_23_ H_29_ N O_6_	438.1904	415.2011	39.356	1.82
3‐*α*‐Hydroxydeoxygedinin	Triterpenoid	C_28_ H_36_ O_6_	469.2604	468.2529	40.373	1.48
Negative mode						
MG(22:6(4Z,7Z,10Z,13Z,16Z,19Z)/0:0/0:0)	Monoglyceride	C_25_ H_38_ O_4_	401.2709	402.2782	43.305	7.71
Catechin‐4beta‐ol	Flavonoid	C_15_ H_14_ O_7_	305.0677	306.075	25.49	7.67
14alpha‐Hydroxyixocarpanolide	Sesquiterpenoid lactone	C_28_ H_40_ O_7_	487.2709	488.2782	39.551	5.19
Aloesol	Aromatic compound	C_13_ H_14_ O_4_	233.0826	234.0899	20.318	4.61
Diosmetin	Flavonoid	C_16_ H_12_ O_6_	299.057	300.0643	30.424	3.93
Trans‐O‐methylgrandmarin	Alkaloid	C_16_ H_18_ O_6_	305.1036	306.1109	28.227	3.72
Verimol C	Terpenoid	C_18_ H_20_ O_4_	299.1297	300.137	43.204	3.64
Salfredin B11	Phenolic compound	C_13_ H_12_ O_4_	231.0669	232.0742	21.096	2.55
3*β*‐Hydroxydeoxodihydrodeoxygedunin	Triterpenoid	C_28_ H_38_ O_6_	469.261	470.2682	36.701	2.20
(2E)‐Piperamide‐C5:1	Alkaloid	C_16_H_19_NO_3_	272.13	273.1373	37.454	1.99

On the other hand, in negative mode, the abundance was found to be less, but the identified compounds were more in types, such as MG (22:6(4Z,7Z,10Z,13Z,16Z,19Z)/0:0/0:0), which was the dominant compound, followed by catechin‐4beta‐ol, 14alpha‐hydroxyixocarpanolide, aloesol, and diosmetin. Compounds like catechin and diosmetin, which are presented as the major compounds in the negative mode of EAE‐P2NS8, are natural flavonoids. Compounds such as catechin and its derivatives have attracted attention for their unique therapeutic effects [51] and are considered potential metabolites with diverse bioactive antibiotic activities, or even nonspecific bactericidal substances, that could eventually inhibit or kill bacteria [53]. Diosmetin, a flavone aglycone and the major metabolite of diosmin, which is a naturally occurring flavonoid glycoside, has shown antioxidant, anti‐inflammatory, antidiabetic, antihyperglycemic, antilipid peroxidative, antimutagenic, antihypertensive, antihyperlipidemic, anti‐arteriosclerotic, anti‐apoptotic as well as antitumor activity [54]. Moreover, diosmetin has been reported to have potential as a new antivirulence drug for *S. aureus* infections, particularly for targeting alpha‐hemolysin, a pore‐forming cytotoxin [55]. In addition, (2E)‐piperamide‐C5:1 was detected in the negative mode. It has been reported as a natural product from *Piper arboreum*, *Piper hispidum*, and *Piper nigrum* [56–58]. However, this compound has never been reported from the endophytic fungi *Fusarium concentricum* isolated from *P. betle*. The antimicrobial activity of piperamide A and B, isolated from *P. betle* leaves, against *Streptococcus mutans*, *Streptococcus sanguinis*, and *Candida albicans* has been documented [59]. This result revealed that endophytic fungi isolated from *P. betle* produced secondary metabolites related to the plant host. Secondary metabolites are produced by endophytic fungi to protect plants from pathogens [60].

### 3.8. Molecular Identification of Endophytic Fungus Strain P2NS8

**Figure 5:**
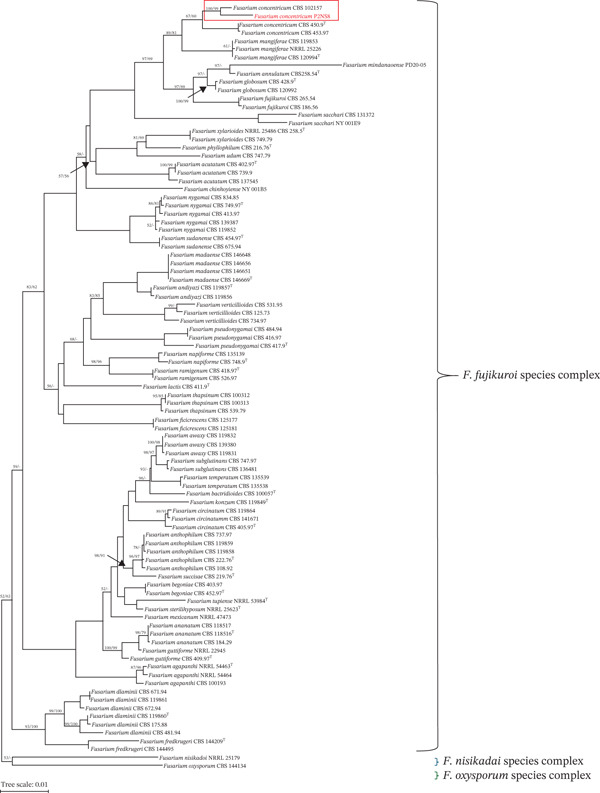
Phylogenetic analysis of *F. concentricum* P2NS8 using five combined loci (*tef1*, *rpb2*, ITS, *tub2*, and *rpb1*). Bootstrap values for maximum likelihood/maximum parsimony (BSML/BSMP) equal to or greater than 50% were put on the node.

The fungal isolates showing the highest activity were subjected to molecular identification using five combined loci (*tef1-α*, *rpb1*, *tub2*, ITS, and *rpb2*) to determine their identity. Based on phylogenetic tree analysis using the MP and ML methods, the endophytic fungus strain P2NS8 belongs to *Fusarium fujikuroi* species complex (Figure 5). The endophytic fungus strain P2NS8 from *P. betle* was grouped with *F. concentricum* CBS 102157, with 100% bootstrap support in ML analyses and 99% bootstrap support in MP analyses. Therefore, the endophytic fungus strain P2NS8 was identified as *F. concentricum.* The sequences of the combined genes *tef1-α*, *rpb1*, *tub2*, ITS, and *rpb2* were deposited in the NCBI GenBank database with accession numbers PP853384, PP858897, PP858898, PP858899, and PP858900, respectively. The identification of complex species of *Fusarium* using the multigene phylogeny, including ITS, *rpb1*, *rpb2*, *tef1-α*, *tub2*, and calmodulin (*CaM*), has been reported [61].

## 4. Conclusion

The endophytic fungus strain P2NS8, identified as *F. concentricum*, demonstrated impressive antimicrobial and antioxidant properties. This strain exhibited both bacteriostatic and bactericidal activity against various pathogenic and spoilage bacteria, including *L. monocytogenes* ATCC 15313, *Shewanella* sp. TBRC 5775, and *S. aureus* ATCC 25923. The EAE‐P2NS8 exhibits various modes of action, including structural damage to bacterial cells, such as cell wall destruction and pore formation, leading to cell lysis. For cytotoxicity against Caco2 cells, EAE‐P2NS8 at low concentrations maintained high cell viability; however, increasing the concentration decreased cell viability, indicating toxicity at high levels. LC‐QTOF‐MS analysis revealed the presence of various flavonoid and alkaloid compounds within EAE‐P2NS8, known for their antimicrobial effects. However, to enhance antimicrobial activity, EAE‐P2NS8 should be used in combination with other commercial food preservative agents to control *L. monocytogenes* ATCC 15313, *Shewanella* sp. TBRC 5775, and *S. aureus* ATCC 25923, while reducing the cytotoxicity of EAE‐P2NS8.

## Author Contributions

**Ankita Singh:** writing – original draft, methodology, investigation, formal analysis, visualization. **Sita Preedanon:** formal analysis, software. **Soottawat Benjakul:** writing – review and editing, validation, resources. **Jirayu Buatong:** writing – review and editing, visualization, validation, supervision, project administration, funding acquisition, data curation, conceptualization.

## Funding

This study was supported by the Reinventing University Program, Ministry of Higher Education, Science, Research and Innovation, Thailand, and the PSU Research Grant for Thesis (Fiscal Year 2025), Graduate School, Prince of Songkla University, Thailand.

## Conflicts of Interest

The authors declare no conflicts of interest.

## Supporting information


**Supporting Information** Additional supporting information can be found online in the Supporting Information section. Table S1: Classification of seven endophytic fungal *Fusarium* spp. isolated from *Piper retrofractum* and *Piper betel* based on morphological characteristics. Table S2: Minimum inhibitory concentration and minimum bactericidal concentration of EAE from four endophytic *Fusarium* species against pathogenic and spoilage bacteria.

## Data Availability

The data that support the findings of this study are available from the corresponding author upon reasonable request.
