# Supplementary material for: Bioactive Potentials of Endophytic Fungus Fusarium concentricum P2NS8 Derived From Piper betel Linn.: Antibacterial, Antioxidant, and Cytotoxic Effects
Source: Int J Microbiol. 2026 May 30;2026:7968494. doi: 10.1155/ijm/7968494 (PMC13239169; doi:10.1155/ijm/7968494)
Supplement: Supplementary file 1 — Supporting Information Additional supporting information can be found online in the Supporting Information section. Table S1: Classification of seven endophytic fungal Fusarium spp. isolated from Piper retrofractum and Piper betel based on morphological characteristics. Table S2: Minimum inhibitory concentration and minimum bactericidal concentration of EAE from four endophytic Fusarium species against pathogenic and spoilage bacteria. [file IJM-2026-7968494-s001.docx]

**Supplementary data**

**Table S1** Classification of seven endophytic fungal Fusarium spp. isolated from Piper retrofractum and Piper betel based on morphological characteristics

| **Code** | **Morphotype group** | **Plant source** |
| --- | --- | --- |
| P1LaS2 | A | *Piper retrofractum* |
| P1LaS7 | A | *Piper retrofractum* |
| P1LaS8 | A | *Piper retrofractum* |
| P2NS4 | B | *Piper betel* |
| P2NS5 | B | *Piper betel* |
| P2NS6 | B | *Piper betel* |
| P2NS8 | B | *Piper betel* |

**Table S2** Minimum inhibitory concentration and minimum bactericidal concentration of EAE from four endophytic *Fusarium* species against pathogenic and spoilage bacteria

| Bacterial strains | MIC/MBC (mg/mL) | | | |
| --- | --- | --- | --- | --- |
|  | P1LaS7 | P2NS8 | P1LaS2 | P2NS4 |
| SP | >4.096/>4.096 | 0.128/0.512 | >4.096/>4.096 | 0.256/0.512 |
| LM | 1.024/2.048 | 0.256/0.512 | 1.024/4.096 | 0.128/0.512 |
| SA | 0.512/1.024 | 0.064/0.256 | 0.512/1.024 | 0.128/0.512 |

SP: *Shewanella* sp. TBRC 5775; LM: *L. monocytogenes* ATCC 15313; SA: *S. aureus* ATCC 25923; MIC: minimum inhibitory concentration; MBC: minimum bactericidal concentration
